# The effect of background and illumination on color identification of real, 3D objects

**DOI:** 10.3389/fpsyg.2013.00821

**Published:** 2013-11-11

**Authors:** Sarah R. Allred, Maria Olkkonen

**Affiliations:** COVI Research Lab, Department of Psychology, Rutgers – The State University of New JerseyCamden, NJ, USA

**Keywords:** color appearance, constancy, illumination, background, object, 3D

## Abstract

For the surface reflectance of an object to be a useful cue to object identity, judgments of its color should remain stable across changes in the object's environment. In 2D scenes, there is general consensus that color judgments are much more stable across illumination changes than background changes. Here we investigate whether these findings generalize to real 3D objects. Observers made color matches to cubes as we independently varied both the illumination impinging on the cube and the 3D background of the cube. As in 2D scenes, we found relatively high but imperfect stability of color judgments under an illuminant shift. In contrast to 2D scenes, we found that background had little effect on average color judgments. In addition, variability of color judgments was increased by an illuminant shift and decreased by embedding the cube within a background. Taken together, these results suggest that in real 3D scenes with ample cues to object segregation, the addition of a background may improve stability of color identification.

## 1. Introduction

For the surface reflectance of an object to be a useful cue to object identity, judgments of its color should remain relatively stable across changes in the object's environment. This stability is known as color constancy. Achieving color constancy between scenes poses a difficult problem for the visual system because the sensory signal that reaches the eye from a scene confounds the surface reflectance of objects within the scene and the illumination impinging on the scene. For example, imagine moving a coffee mug from the kitchen counter to a patio table outside. Both the illumination and the sensory signal reaching the eye from the mug and the area surrounding it will change. The reflectance properties of the mug have not changed, but the reflectance properties of its surrounding surfaces have. The challenge for the visual system is to correctly parse the changing sensory signal in a fashion that supports color identification.

A complete theory of color vision would characterize behavior in real-world color tasks for objects in scenes where both the illumination and surrounding objects change. We are still far from this goal, both because the characterization of such realistic stimuli is currently a computationally intractable problem and because typical laboratory tasks diverge from real-world tasks in a number of ways (see Brainard and Radonjic, in press, for discussion).

There are several approaches available as we seek to move toward a more complete theory of color vision. One general approach is to simplify from the complexity of realistic stimuli and tasks to more carefully controlled tasks and stimuli, with the goal of uncovering principles that govern the relationship between stimuli, task, and color judgments. The hope is that such principles will generalize well to more complex tasks and stimuli. Experiments in this vein have achieved success in demonstrating relationships between early physiological mechanisms and judgments of color appearance (Werner and Walraven, 1982; Webster and Mollon, 1994; Engel and Furmanski, 2001) and have guided the development of computational models that can predict color appearance judgments (McCann et al., 1976; McCann, 2004). However, an important question is whether such principles in fact generalize to color judgments of more realistic stimuli and in more ecologically relevant tasks. Indeed, recent work highlights the difficulty of linking early physiological mechanisms to the later cortical mechanisms that presumably underlie functional color judgments in complex scenes (Gegenfurtner, 2003; Solomon and Lennie, 2007; Witzel and Gegenfurtner, 2013). Thus, a complementary class of experimental approach is to measure color judgments that employ more realistic tasks and stimuli. Because the critical variables underlying perception of such realistic stimuli are not yet amenable to a clear computational characterization, this approach has the disadvantage that the data are not obviously applicable to known physiological mechanisms and models. However, such experiments can provide important guidance about ecologically relevant variables as we develop increasingly complex models of human color vision.

Here we take the second approach with the goal of measuring color judgments in 3D scenes with a real-world color task. In the remainder of the introduction we outline the principles that might be expected to generalize from simpler scenes to govern such color judgments.

In many cases it is now possible to predict successfully color judgments of a uniformly illuminated flat test stimulus surrounded by other flat stimuli. For example, one can start with the responses of cones in the retina, and compute color estimates explicitly using computations grounded in the opponent chromatic and luminance responses of cells early in the visual system (Land and McCann, 1971; McCann et al., 1976; McCann, 1992a; Zaidi et al., 1992; Nayatani, 1997). Although a vigorous debate continues about the exact mapping between local contrast and color appearance (McCann, 1992b; Singer and D'Zmura, 1994; Brown and MacLeod, 1997; Zaidi et al., 1997; Blakeslee and McCourt, 2001; Rudd and Zemach, 2004; McCann, 2006; Ekroll and Faul, 2012), local contrast in some form is central to many theories. Such local contrast mechanisms in principle support color constancy under illumination shifts, but yield poor color constancy under background shifts. Consistent with this, a large body of work suggests that color constancy in 2D scenes is relatively high under illuminant shifts (Smithson, 2005; Shevell and Kingdom, 2008; Foster, 2011; Brainard and Radonjic, in press) but relatively poor under background shifts (McCann, 1992b; Kraft et al., 2002; Werner, 2006).

As we move from 2D scenes with uniform illumination to 3D scenes with non-uniform illumination, an important question is whether these consistent findings of high constancy under illumination shifts and poor constancy under background shifts will generalize. There are at least two reasons to be cautious about such generalizations.

First, as scenes become more complex, the local contrast relationships between object and background likewise become more complex. For example, the light reaching the eye from an object of one surface reflectance may vary because object pose with respect to the illuminant introduces illumination gradients or shadows, because of variation in the illumination itself, because of the texture of the object, or because of specular highlights (Brainard and Radonjic, in press).

Thus far, the empirical research is mixed. In support of generalization, some recent work suggests that, as in 2D scenes, observers adjust color matches to compensate partially for illumination gradients (Boyaci et al., 2003, 2004; Ripamonti et al., 2004; Allred and Brainard, 2009; Xiao et al., 2012). Also as in 2D scenes, constancy is less stable when the surfaces surrounding an object change than when the illuminant changes, and constancy is particularly poor when both are manipulated together (Delahunt and Brainard, 2004; Allred and Brainard, 2009). Similarly, Kraft et al. (2002) found that reducing cues to depth in a real scene had little effect on color constancy, suggesting that at least in some cases, depth is not a critical variable.

In contrast, other research suggests a more complicated picture. For example, (Xiao et al., 2012) reported interactions between illuminant cues and object form, and perceived color can be strongly influenced by the perceived shape of a test stimulus (Adelson, 1993; Bloj et al., 1999) or the region of the scene with which a test stimulus is perceptually grouped (Gilchrist, 1977; Schirillo and Shevell, 2000). And it is clear that the geometric structure of a scene can exert effects on color judgments beyond those that can be explained by local contrast. For example, Radonjić and Gilchrist (2013) found that perceived depth modulates perceived lightness even when local luminance ratios remain constant, and Werner (2006) demonstrated that the addition of depth cues alone improves color constancy.

A second reason to be cautious about generalization is that the task facing the observer may also be complicated by increasing scene complexity. For example, consider again the mug moved from inside to outside. An observer might notice subtle differences in the appearance of the mug—one surface might appear shadowed, for example—while simultaneously recognizing that the reflectance properties of the mug itself are uniform and unchanged from indoors. Although observers may make distinct appearance and reflectance judgments in 2D scenes (Arend and Reeves, 1986; Troost and de Weert, 1991; Arend and Spehar, 1993a,b; Bäuml, 1999; Blakeslee and McCourt, 2001), the greater physical complexity of 3D scenes may exacerbate those distinctions. Many previous studies in 2D scenes either explicitly or putatively rely on proximal or appearance judgments (Brainard and Radonjic, in press). However, many real-world color tasks require us to identify objects between scenes rather than make exact appearance matches (Zaidi, 1998; Abrams et al., 2007). For example, when picking out a thread at the store to match a button at home, we seek to match the reflectance properties of thread and button, not the color appearance between home and store illumination. Thus, to the extent that observers make reflectance rather than appearance judgments in 3D scenes, results from 2D scenes may fail to generalize. We do note that the literature surrounding appearance and reflectance instructional effects is somewhat murky (Brainard et al., 1997; Blakeslee and McCourt, 2001; Ripamonti et al., 2004; Allred and Brainard, 2009; Allred, 2012; Brainard and Radonjic, in press), and we return to this topic in the discussion.

To summarize, here we measured color identification of real 3D objects. Observers made color matches for real cubes presented in an unevenly illuminated three-dimensional scene in which we independently manipulated both the illumination impinging on the scene and a three-dimensional background in which the cube was embedded. To examine real-world task constraints, observers matched the reflectance of the object.

## 2. Materials and methods

Observers were 122 college students who participated for course credit. All experimental procedures were approved by the Rutgers IRB (Protocol #E10-410) and written informed consent was acquired from all observers. Observers had normal or corrected-to-normal visual acuity and normal color vision as assessed by the Ishihara Color plates. Observers entered a room and viewed two adjacent 4′ × 4′ × 4′ gray flat matte booths. Illumination in the room was provided separately for each booth (chromaticity in CIE uvY space; Booth A: *u* = 0.27, *v* = 0.53, CCT ~2600K; Booth B: *u* = 0.22, *v* = 0.50, CCT ~4000K).

**Figure 1:**
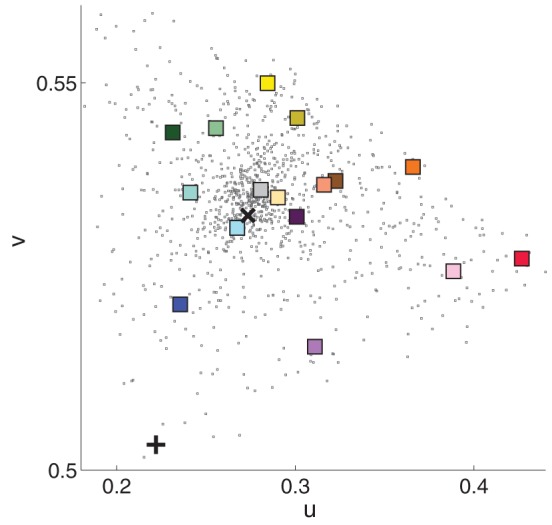
**Chromaticity in CIE uv coordinates of the 16 cube stimuli (colored squares), 1022 paint chips (black dots), and illumination for Booth A (black x) and Booth B (black +)**. Luminance information was discarded. Plotted measurements were made in Booth A. The square's color is an approximation of the cube's apparent color.

**Figure 2:**
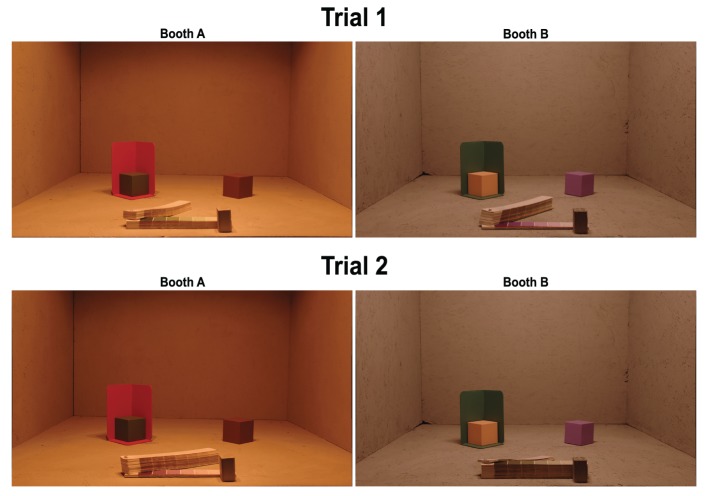
**Photograph of experimental setup for one example trial**. On each trial, observers viewed four cubes, two cubes each in Booth A (left images) and Booth B (right images) that were separately illuminated. On each trial, one cube in each booth was embedded in a 3D background (for this condition, left cubes in each image). The matching palette (booklet in the front of each booth) contained 1022 paint chips. The palette in each booth rotated freely on a long screw mounted into palette, and the wooden stopper prevented observers from pulling palette strips out of the booth. Observers were permitted to flip freely through the book, but were instructed to choose a match only when the palette strip was aligned with the stopper. On Trial 1 (baseline and background conditions) observers chose color matches from the palette mounted in the same booth as the cubes. To illustrate this, the palette is open to the green section (Trial 1, Booth A) and the purple section (Trial 1, Booth B). On Trial 2 (illumination and joint conditions) observers chose color matches for a cube from the palette mounted in the other booth. As illustrated, the color match for the green cube (Booth A) was selected from the palette in Booth B, and the color match for the purple cube (Booth B) was selected from the palette in Booth A.

Observers sat in a rolling chair and were free to move positions. Mounted 4.5″ from the front of each booth was a book of 1022 commercial paint chips (Sherwin-Williams, 2010) which served as a matching palette (Figure 2). The palette mount allowed observers to rotate individual palette strips into the booth, but a stopper prevented the palette strips from rotating out of the booth illuminant. Each palette strip contained either 7 or 8 paint chips. Experimenters monitored observers to make sure that they did not climb into the booths or move the cubes. Observers were instructed in each condition to choose the paint chip that matched the paint of the cube under study, and observers were instructed to make their final chip selection when the palette strip was aligned with the stopper (see Figure 2). The instructions were intended to evoke reflectance rather than appearance matches. Sixteen 3″ × 3″ × 3″ cubes (subtending 4.5°–6.5° at usual viewing distances), painted with different colors of flat matte paint chosen to approximately span color space (see Figure 1) served as stimuli.

Observers made color matches by inspecting the paint palette and writing the number corresponding to the paint chip that best matched the paint on the cube. In the *baseline* condition, which served as the comparison for all other conditions, observers viewed the cubes and matching chips in the same booth (Figure 2, Trial 1, right cubes). In the *illumination* condition, observers looked between booths while viewing cubes in one booth and matching chips in the other booth (Figure 2, Trial 2, right cubes). The *background* condition differed from the that the cube was embedded in a three-dimensional background (Figure 2, Trial 1, left cubes). The *joint* condition combined manipulations, so that cubes were embedded in the background in one booth and the matching chips were viewed in the other booth (Figure 2, Trial 2, left cubes).

**Figure 3:**
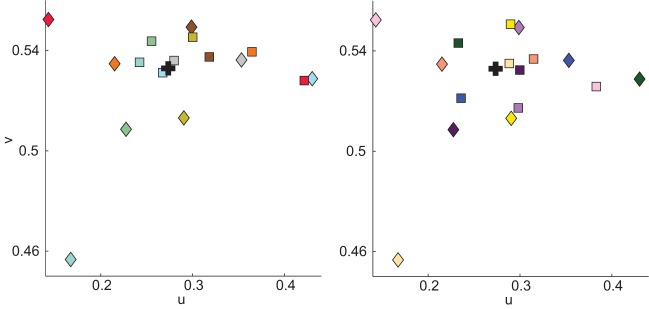
**Illustration of cube (squares) and background (diamond) chromaticity as measured in Booth A**. Stimuli are divided into panels to aid visualization. Square color indicates the apparent color of the cube; diamond color illustrates the apparent color of the cube with which the background was paired and not the apparent color of the background. Color specifications for cubes and backgrounds are in Table 2. Black + indicate illuminant chromaticity.

Each observer performed two trials (see Table 1), one each on two different days. On each trial, observers viewed four different cubes, two in each booth. One cube in each booth was embedded in a background (see Figure 2). This yielded eight color matches per observer, two in each condition. Thus, each observer made color matches for 8 of the 16 cubes. On Trial 1, color matches were made from the palette mounted in the booth in which the cube was viewed (baseline and background conditions) and on Trial 2, color matches were made from the palette in the opposite booth (illumination and joint conditions, see Table 1). To prevent order effects, we counterbalanced between observers to achieve color matches for each cube in each of the four conditions; thus, observers never viewed an individual cube in more than one condition. We did not counterbalance the booth in which cubes were seen; thus, in the illumination condition, half the cubes were viewed in Booth A and matched in Booth B, and the other half were viewed in Booth B and matched in Booth A (see Table 2). There were a total of eight different backgrounds. Each cube was seen with only one background. Background paints were chosen by eye to be approximately color-opponent while remaining in a different color category from any other stimulus (cube or background) present within a particular trial. The category restriction sometimes resulted in non-opponent color pairings. The color categories and chromaticities for each cube and its background are enumerated in Table 2, and illustrated in Figure 3. Implications of cube/background pairings are addressed in the discussion.

**Table 1:** **Trial description**.

**Trial 1**			**Trial 2**		
**Cube**	**Condition**	**Booth see/match**	**Cube**	**Condition**	**Booth see/match**
Loc 1	Baseline	A / A	Loc 1	Illumination	A / B
Loc 2	Background	A / A	Loc 2	Joint	A / B
Loc 3	Baseline	B / B	Loc 3	Illumination	B / A
Loc 4	Background	B / B	Loc 4	Joint	B / A

*On each trial, observers viewed four cubes in different locations (left column). The cubes are placed in these four locations (from left to right) in Figure 2. The second column indicates the condition of each cube. The third column indicates the booth where the cube was viewed (See) and the booth in which the color match was chosen from the paint palette (Match). Each cube was viewed in only one location. To achieve color matches for each cube in each condition between observers, we counterbalanced the location of the background and the trial (1 or 2) in which the cube was presented*.

**Table 2:** **Description of 16 cube stimuli (left half) and backgrounds (right half)**.

	**Cube name**	**Booth**	**u**	**v**	***cd/m*^2^**	**Background name**	**u**	**v**	***cd/m*^2^**
1	Ice blue	A	0.27	0.53	62.54	Red	0.43	0.53	14.27
2	Dull green	A	0.25	0.54	21.77	Dull blue	0.23	0.51	7.19
3	Orange	B	0.36	0.54	45.06	Surf green	0.22	0.53	12.01
4	Dark brown	B	0.32	0.54	16.61	Yellow	0.30	0.55	58.64
5	Dark green	A	0.23	0.54	13.74	Red	0.43	0.53	14.27
6	Plum	A	0.30	0.53	13.48	Dull blue	0.23	0.51	7.19
7	Peach	B	0.31	0.54	59.50	Surf green	0.22	0.53	12.01
8	Purple	B	0.30	0.52	28.01	Yellow	0.30	0.55	58.64
9	Gold	A	0.30	0.54	41.82	Purple	0.29	0.51	18.47
10	Aqua	A	0.24	0.53	67.15	Hyper blue	0.17	0.46	3.62
11	Gray	B	0.28	0.54	48.80	Peach	0.35	0.54	32.38
12	Red	B	0.42	0.53	19.93	Green	0.14	0.55	5.62
13	Yellow	A	0.30	0.55	71.38	Purple	0.29	0.51	18.47
14	Doeskin	A	0.29	0.53	48.75	Hyper blue	0.17	0.46	3.62
15	Secure blue	B	0.24	0.52	19.69	Peach	0.35	0.54	32.38
16	Pink	B	0.38	0.53	36.15	Green	0.14	0.55	5.62

Names refer to the apparent color of the cubes and backgrounds. Each row gives one cube/background pairing. Cubes were always paired with the same background. Radiometer measurements are under Booth A illumination. Booth indicates where the cube was seen (see Table 1).

Color specifications were made using a Spectrascan PR-655 spectral radiometer (Photo Research Inc., Chattsworth, CA). Conversions between color spaces (wavelength to CIE uvY) were made using standard equations implemented in Matlab's Psychophysics Toolbox (Brainard, 1997). The white point was taken as the illuminant, measured with a reflectance standard (PhotoResearch, Inc. RS-2, Mg0 standard). In all analyses, we discard luminance and use only chromaticity values.

To specify the cube chromaticity, we measured each cube in the location where it was experimentally presented. The radiometer was positioned to approximate the average observers' eye point; however, there is considerable variability in this eye point since observers ranged in height and were free to move outside the booths. For each cube, measurements were from the top surface of the cube, in the corner closest to the observer. Repeat measurements were taken over the course of the experiment and showed very small deviations in chromaticity and somewhat larger variations in luminance. Chromaticity measures of the background were made on the bottom surface of the background closest to the observer, nearly below the location of the cube measurement. Radiometer measurements for each paint chip in each booth were made near the center of the paint chip. Although each cube and each background were painted uniformly, the 3D structure of the scene elicited considerable variations in luminance across each surface. This variation is seen easily in Figure 2. In this experiment, we made no attempt to control for or manipulate luminance. Radiometer measurements confirmed that chromaticity was relatively stable across surfaces.

### 2.1. Data analysis

We discarded data from 11 of 122 observers for failure to understand the task as indicated by not recording a response for more than half the cubes, or for systematically recording cube color in the wrong location. From the remaining 888 trials (111 observers × 8 cubes), we discarded a further 105 trials for the following reasons: indecipherable or non-existent card notation (82/888 trials, 9%), missing radiometer data (7/888 trials, <1%) or color match of a clearly different, non-adjacent color category (15/888, 2%). To determine which matches fell into the last group, two lab members independently examined each color match and rated it as either within normal limits or of a clearly different category. Lab members were provided a list of matches for each cube but were not informed about the condition in which the match was chosen. Only matches judged as the wrong category by both lab members were discarded. In most cases (12/15), the discarded match seemed to match another cube on that trial, and thus probably reflects an observer recording the paint chip in the wrong location. For example, observers recorded a pink match for the blue cube and a blue match for the pink cube. Overall, similar numbers of color matches were discarded in each condition: baseline (21), illumination (30), background (26), joint (27).

In all cases where significance levels are reported for a family of statistical tests, we report the *p*-value without the Bonferroni correction. We do so because the assumptions underlying the uncorrected *p*-value are relatively transparent, whereas the criteria for including a test within a specific family are not always clear.

#### 2.1.1. Color constancy index

Many different metrics are used to describe color constancy (e.g., Foster, 2011). We described color matches across an illuminant shift by computing a color constancy index based on a modified-Brunswick ratio (*mBR*). This index describes the extent to which observers alter the chromaticity of color matches in the direction expected by color constancy. Values near 1 indicate high color constancy, such that observers selected a paint chip with chromaticity equal to that of the cube measured under the matching illuminant. Values closer to 0 indicate failure to compensate for the illuminant shift, and values of greater than 1 indicate overcompensation for the illuminant shift. We calculated the *mBR* as follows:

(1) $$mBR=\left(\frac{proj_{p\vec{h}ys}p\vec{e}rc}{∥p\vec{h}ys∥}\right)$$

where $p\vec{e}rc$ is the perceptual shift caused by the illuminant shift, taken as the average color match in the illumination condition. In this index, $p\vec{h}ys$ is the chromaticity of the illuminant shift. Calculating $p\vec{h}ys$ is non-trivial; the illumination impinging on the cube in both booths is non-uniform, both because of the location of the illuminant and the 3D structure of the cubes. This is seen clearly in Figure 2, where the top of the cube reflects more light than the sides. Because $p\vec{h}ys$ varies across the booth, and because we had no way of knowing which portions of the cube the observers utilized for their matches, we calculated $p\vec{h}ys$ as follows: First, we made the assumption that the area of the booth observers utilize in making color matches is independent of condition. If this is a secure assumption, then a perfectly color constant observer would pick the same palette chip as a match in the overall and joint conditions as in the baseline condition. We took the palette chips chosen in the baseline condition, measured their chromaticity in the illumination condition, and took this as $p\vec{h}ys$. Both $p\vec{e}rc$ and $p\vec{h}ys$ require a reference chromaticity. For the reasons just described, the reference was defined as the chromaticity of the average match in the baseline condition, rather than the chromaticity of the cube measured under the baseline illuminant. Thus, the constancy indices as calculated here are best described as relative constancy indices: the *mBR* measures the concordance between color matches in the baseline condition and color matches in each experimental condition, rather than the concordance between color match chromaticity and cube chromaticity.

#### 2.1.2. Error index

To compare directly constancy in the illumination and background conditions, it would be useful to have a measure of constancy in the background condition. However, such a constancy index requires a definition of what constitutes a failure of constancy. In simple 2D scenes, one can estimate these failures using algorithms that equate cone contrasts between the baseline and background conditions. It is less obvious how such algorithms should be applied to our 3D stimuli, both because the cone contrast between cube and background varies substantially with scene location, and because we lack an empirical characterization of which parts of a 3D scene should be incorporated.

Thus, to avoid subscribing to a particular theoretical approach, we chose a relatively atheoretic error index (*eI*) to compare matches in the baseline and experimental conditions. To compute the *eI*, we took the distance in color space between the average color match and the color constant match, as described above. We defined the *eI* in the baseline condition as the split-half error, calculated by randomly dividing the baseline data into two groups and computing the distance between the average color match in each of the two groups.

#### 2.1.3. Central tendency

We characterized the average color match of the data in each condition in two ways: First, after discarding luminance information, we took the mean u and v chromaticites across all matches in a condition as the average color match. Second, we determined the ellipse that best-fit the color matches in a least-squares sense (Fitzgibbon et al., 1999), and used the center of the ellipse as a measure of the average color match. The pattern of results is qualitatively the same with both measures of central tendency. Here we report the mean chromaticity as the average color match.

## 3. Results

The main goal of this paper is to investigate the effect of illumination and background shifts on color matches. To that end, we first show color matches for all observers for representative individual cubes, and then turn to quantitative comparison across all cubes.

### 3.1. Individual cubes

**Figure 4:**
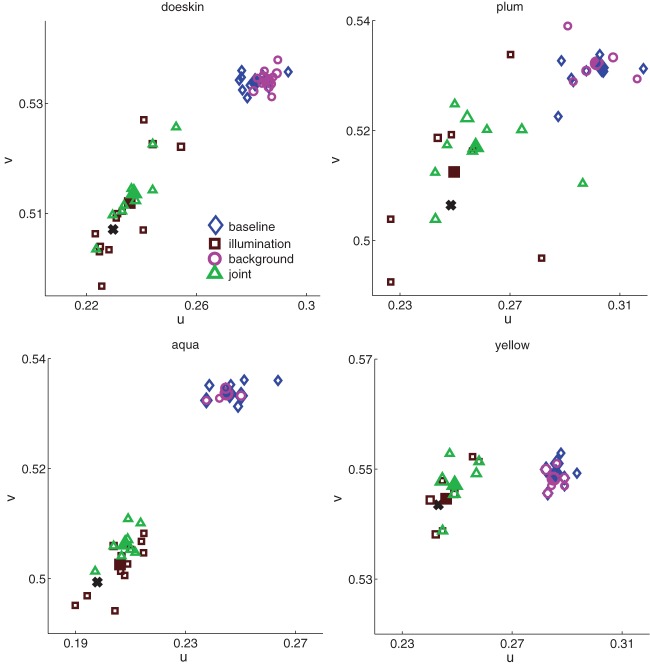
**Color matches in all four conditions (baseline, blue diamonds; background, magenta circles; illumination, brown squares; joint, green triangles) for four of the sixteen cubes**. Each unfilled data point represents one paint chip chosen; the size of the data point represents the number of observers who chose that chip. Filled data points represent average color matches, and black solid crosses show constancy predictions. The x- and y-axis ranges are consistent between plots, though the starting point shifts to accommodate the relevant chromaticity range.

Color matches for all observers and all conditions for four of the sixteen cubes are shown in Figure 4. From these plots, several salient points can be made.

First, in the baseline condition, observers chose many different paint chips (unfilled blue diamonds, Figure 4). This range of color matches in the baseline condition was a common feature across all cubes (median number of paint chips chosen in baseline condition = 7, min = 4, max = 10; median number of observers per cube = 12). The trend of variability in baseline color matches is reassuring. The basic task employed here, choosing a flat paint chip from a commercial palette book to match a three-dimensional cube located at a distance from the palette, is somewhat non-traditional. Thus, the baseline data provide a useful sanity check: the paint palette was sufficiently discretized to provide a reasonable estimate of between-observers variability in color perception.

Although observers chose many paint chips for each cube, the region of color space spanned by the individual matches varies between cubes. For example, the paint chips chosen for plum in the baseline condition span a larger region of color space than do the chips chosen for aqua, yellow and doeskin. These differences could reflect true differences in color perception between cubes, or they could reflect the non-uniformity of the paint chips in color space seen in Figure 1. For the moment, we do not attempt to disentangle inherent inhomogeneities in color perception between cubes from palette inhomogeneities; rather, we seek in the subsequent analyses to ask how background and illumination affect color matches for a given cube.

Second, for each cube shown, observers exhibited relatively high but imperfect degrees of color constancy under a change in illumination. If observers were perfectly color constant; that is, if the observers chose the same paint chips under the illuminant shift as they did in the baseline condition, then individual data points (brown squares) would cluster near the constancy prediction (see Materials and Methods) indicated by the black crosses (Figure 4). If, on the other hand, observers matched the sensory signal reaching the eye in the baseline condition and failed to account for the change in illumination, the brown squares should overlap the color matches in the baseline condition (blue diamonds). Most of the brown squares are shifted toward the black crosses, but not identical to them, indicating that observers showed high but imperfect color constancy. Again, observers showed considerable variability in the number of distinct paint chips chosen (median number of paint chips chosen = 8, min = 5, max = 12).

Third, embedding the cubes in a background had little effect on color matches (magenta circles in Figure 4), in contrast to the relatively large effect elicited by a change in the illumination. In most panels, the matches made when the cube was embedded in a background were nearly identical to the blue diamonds of matches made to the cubes in the baseline condition.

Fourth, combining the addition of the surround with an illumination shift seems to have an effect similar to that of the illumination shift alone (green triangles similar to brown squares in Figure 4).

Lastly, inspection of the four panels reveals considerable variability in the extent of color space spanned by individual matches in a given condition. For example, the region of chromaticity space spanned by paint chip choices for the plum cube in each condition seems larger than for the yellow cube. Additionally, for each cube, the region of chromaticity space spanned by the brown and green symbols (illumination and joint conditions) seems larger than the region of chromaticity space spanned by the blue and magenta symbols (baseline and background conditions).

In the remainder of the paper, we quantify the extent to which the effects of experimental condition on both average color constancy and variability noted in the individual panels in Figure 4 are consistent in the entire dataset.

### 3.2. Average color constancy

**Figure 5:**
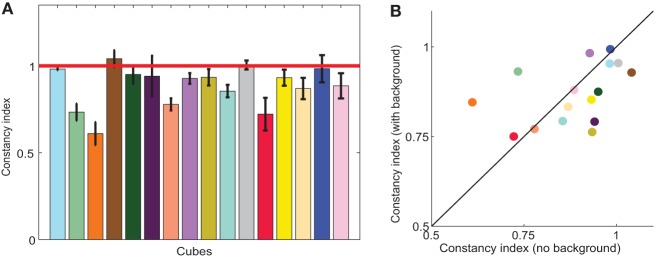
**(A)** Color constancy indices in the illumination condition. Each bar shows the average color constancy index for one cube, with perfect constancy indicated by the red horizontal line. Indices are modified-Brunswick ratios (see Materials and Methods) averaged across observers. Error bars are s.e.m across observers; the average number of observers per cube was 12. **(B)** Average constancy indices in the joint condition (with background; y-axis) and the illumination condition (no background; x-axis). Black diagonal line indicates no effect of background. Bar height **(A)** and symbol color **(B)** approximate apparent cube color.

As with the data for the individual cubes (Figure 4), average color constancy across all cubes under an illumination shift, shown in Figure 5, was generally high but imperfect. To quantify the degree of constancy, it is standard to compute a color constancy index. Such indices seek to frame the data with respect to their position between the *constancy* and *no-constancy* predictions, where 1 indicates perfect constancy, 0 indicates a complete failure of constancy, and indices greater than 1 indicate that observers overcompensated for the illuminant shift. From the constancy predictions (illustrated for the four cubes in Figure 4), we computed such an index (see Materials and Methods). Briefly, the color constancy prediction was derived using the assumption that color constant observers would choose the same paint chips in the baseline condition as in the illumination condition; that is, their matches would reflect consistency in surface reflectance, rather than chromaticity.

Consistent with other color constancy studies of illumination changes in relatively realistic scenes, color constancy indices were quite high, averaging 0.88 ±0.03. Constancy indices are displayed for all cubes in Figure 5A. Although average constancy indices were relatively high, there was substantial variability between cubes, reflected in the varying bar heights in Figure 5A. Indices ranged from 0.61 (orange) to 1.04 (brown). Within a cube, indices were relatively consistent between observers, where the standard error averaged about 6% of the constancy index.

How does embedding a cube in the background affect color matches? The individual data suggest that the effect of background is small. To compare illumination and background conditions, it would be useful to calculate a background constancy index that frames the data between the constancy and no-constancy predictions. However, since we lack a complete characterization of both the theory and low-level computations involved in color constancy in three-dimensional scenes, it is not obvious how to compute the no-constancy prediction for the background condition. In scenes that consist of uniformly illuminated flat stimuli embedded in backgrounds, a simplifying assumption that is based on early processing in the visual system is that a matching surface will appear the same as a study surface when the cone-excitation ratio between the match and its surround equals the cone-excitation ratio between the study surface and its background. Although we computed such local-contrast predictions (not shown), their dependence on luminance meant that the the no-constancy match varied substantially depending on what radiometer measurements were utilized.

To avoid potentially spurious relationships that might either hide or exaggerate the effect of the background, we compared illumination and background matches to baseline matches using a less theoretically motivated error index (*eI*). We defined the *eI* as the distance in color space between the chromaticity of the average match and the constancy prediction. Unlike a constancy index, the eI compares the magnitude of experimental effects and is agnostic about cause or directionality of effects. Such an index is particularly useful in the background condition, where a color constancy index may be influenced heavily by theoretical assumptions and there is less consensus about the size or direction of expected effects.

**Figure 6:**
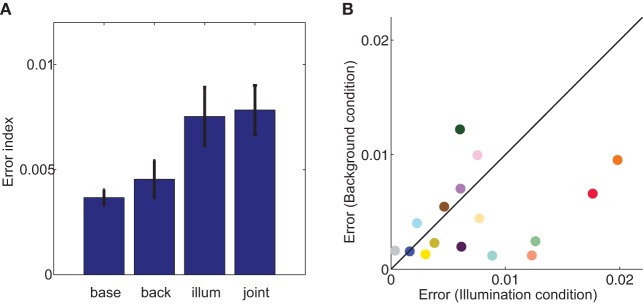
**Error index (*eI*) in each of the four labeled experimental conditions (A) and cube-by-cube comparison in the illumination (x-axis) and background (y-axis) conditions (B)**. In the background, illumination, and joint conditions, the *eI* is defined by calculating the distance in color space between the chromaticity of the color constancy prediction and the actual average chromaticity of paint chips chosen in that experimental condition. In the baseline condition, error is split-half: paint chips for each cube were randomly divided into two groups, and error was defined as the distance in chromaticity space between the means of the two groups. Error bars are s.e.m. across cubes. In **(B)**, symbol color approximates apparent cube color, and black diagonal line is the identity.

For a majority of cubes, errors in the background condition were smaller than errors in the illumination condition, as evidenced by the majority of points being below the identity line in Figure 6B. Aggregated across cubes, this difference was significant (paired two tailed *t*-test, *p* < 0.05; second and third bars, Figure 6A). To provide context for the size of these errors, we compared them to a split-half baseline error (first bar, Figure 6A). Although illumination errors were significantly different than baseline (paired two tailed *t*-test, *p* < 0.05), errors elicited by the addition of a background were no different than baseline errors (paired two tailed *t*-test, *p* = 0.43). Thus, background errors were comparable in size to the variability within the baseline data. Thus, in contrast to the robust phenomenon of color induction in flat stimuli with uniform surrounds, embedding a cube in a background has little effect on color judgments.

Next we asked how the effects of background and illumination combine. Real world color constancy tasks often involve both changes in surrounding surfaces and changes in the illumination, and previous research has suggested that constancy is particularly poor when both changes are made simultaneously (Delahunt and Brainard, 2004). Although we found little effect of embedding cubes in backgrounds without an illuminant shift, it remains possible that there is an interaction between background and illumination.

However, we found that constancy indices were no different in the joint condition than in the illumination condition (two-tailed, paired *t*-test, *p* = 0.57), as demonstrated in Figure 5B, where color constancy indices remained close to the diagonal. The variability of color constancy between cubes in the joint condition was similar to the baseline condition (range with background 0.75–0.99; range without background 0.61–1.04) and marginally correlated (*r* = 0.45, *p* = 0.082) between conditions. Similarly, error indices in the joint condition were no different than in the illumination alone condition (two tailed paired *t*-test, *p* = 0.82, third and fourth bars in Figure 6A). Further consistent with the idea that background elicits no more errors than the baseline condition and the illumination shift elicits the same pattern of errors with or without a background, a Three-Way ANOVA showed no main effect of background, a main effect of illumination and no interaction between illumination and background (Table 3).

**Table 3:** **ANOVA for errors in color matches**.

**Source**	***df***	***F***	***p***
Cube	15	1.72	0.08 (n.s.)
Illumination	1	14.19	<0.001
Background	1	0.39	0.54 (n.s.)
Interaction (ill-back)	1	0.09	0.77 (n.s.)
Error	45		
Total	63		

The table shows the results of a Three-Way ANOVA on the errors for color matches averaged across observers for each cube in each experimental condition. Cube was coded as a random effects variable, while surround and illumination were coded as fixed effects. The ANOVA modeled all main effects and the surround by illumination interaction.

### 3.3. Variability in color matches

In addition to average color matches, we also investigated the effect of background and illumination on the variability of color matches. In all conditions, observers chose a variety of paint chips as color matches (see Figure 4). For each experimental condition, we defined variability as the distance between each color match and the average color match in that condition. Thus, cubes with matches that span a larger region of color space have higher variability.

**Figure 7:**
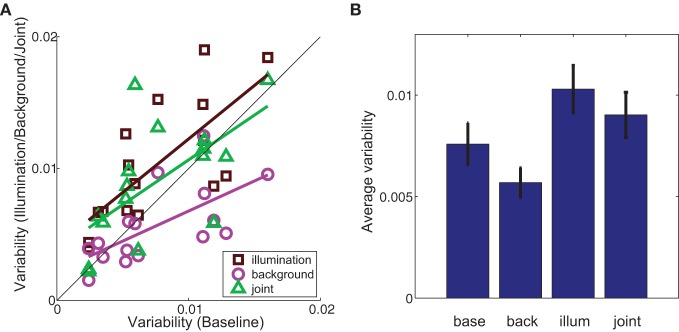
**(A)** Variability of color matches in the illumination (brown squares), background (magenta circles) and joint (green triangles) conditions (y-axis) as a function of variability in the baseline condition (x-axis). Colored lines are best-fit to data in the least-squares sense. Black diagonal line indicates identity. **(B)** Variability in the labeled experimental condition averaged across cubes. Error bars are s.e.m. across cubes (*n* = 16). In both **(A)** and **(B)** variability was defined as the average distance in color space space between each observer's match and the average chromaticity of matches in that condition.

We compared variability in the baseline condition to variability in each experimental condition (Figure 7). If the basic processes underlying color matching are not altered by either the illumination shift or the addition of a background, then the data should fall along the identity line. However, we found that variability in the illumination condition was significantly different than in the baseline condition (brown squares above the line in Figure 7A, two-tailed paired *t*-test, *p* < 0.005). In contrast, adding a background significantly decreased the between-observers variability in color matches (magenta circles below the line in Figure 7A; two-tailed, paired *t*-test, *p* < 0.05).

As with average color matches, cubes within an experimental condition elicited a wide range of variability in color matches. Given the non-uniformity of the palette chip chromaticities in color space, we cannot distinguish whether variability between cubes within a given condition results from increased perceptual variability for that particular color or the non-uniformity with which paint chips sample color space. However, within-cube variability was highly correlated between experimental conditions (baseline-illumination: *r* = 0.73, *p* < 0.005; baseline-background: *r* = 0.65, *p* < 0.01), as it was with color constancy indices, indicating that this variability is related to some property of the cube or palette itself and is not an artifact of differences between observers.

### 3.4. Relationship between average color matches and variability

**Figure 8:**
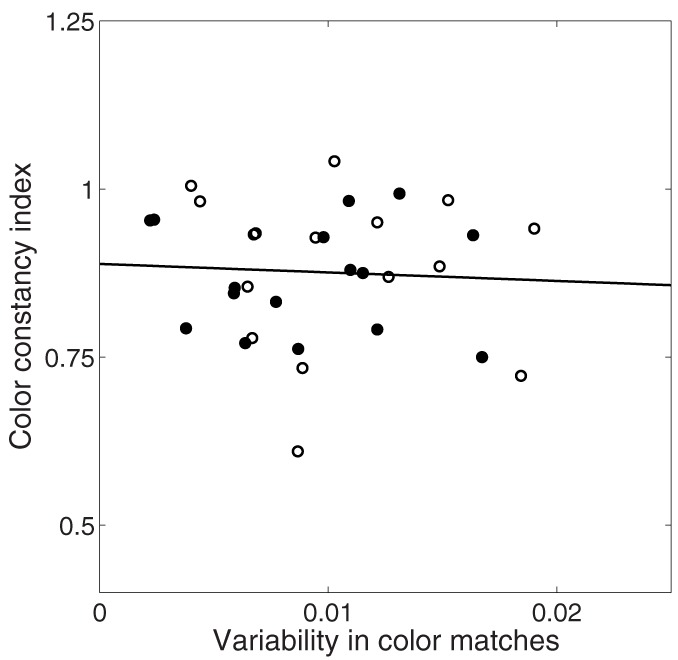
**Relationship between variability in color matches (x-axis) and color constancy index (y-axis) in illumination (unfilled circles) and joint (filled circles) conditions**. Solid black line is best fit line through all data.

Here we have separately analyzed average color matches and variability of color matches, but it is possible that both judgments arise from a common representation. We investigated their independence by plotting variability of matches as a function of color constancy in Figure 8. If, for example, increased variability necessarily led to decreased constancy, we would expect a negative correlation. If, on the other hand, color constancy and variability were unrelated, we would expect no correlation. There was no significant correlation between variability of color matches and degree of color constancy in either the illumination (*r* = −0.10, *p* = 0.71) or joint condition (*r* = −0.03, *p* = 0.91).

A related question is whether the palette non-uniformity is related to within-condition constancy or variability. The between-condition experimental effects are unlikely to be artifacts of palette non-uniformity; for example, the palette discretization is the same for the orange cube in the baseline condition and in the illumination condition. However, of interest is whether the degree of constancy or variability within a condition is predicted by palette density. For example, can palette density account for the relatively high color constancy and low variability of doeskin compared to plum in the illumination condition (Figure 4)?

**Figure 9:**
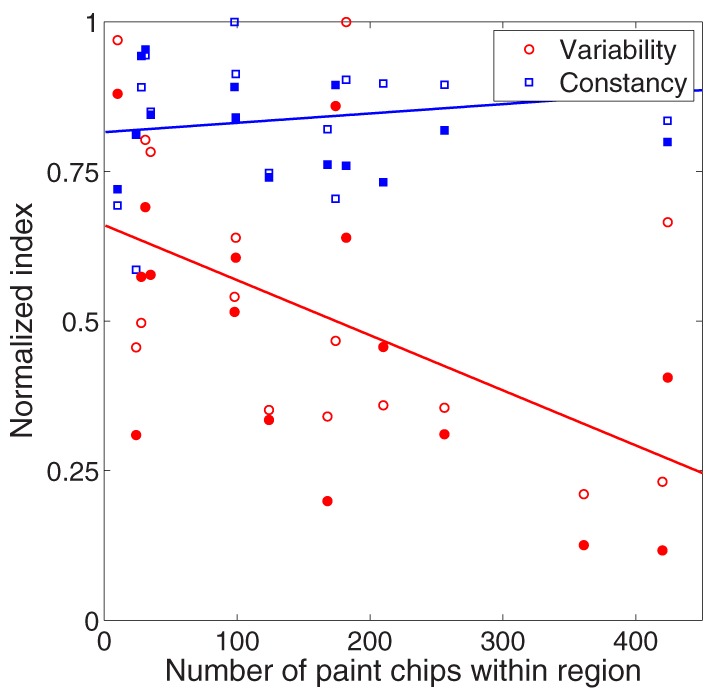
**Relationship between palette density (x-axis) and color constancy (blue symbols, y-axis) and variability (red symbols, y-axis) in the illumination (unfilled circles) and joint (filled circles) conditions**. To aid in visualization, color constancy indices (red symbols) and variability values (blue symbols) were normalized to their respective maxima. All statistical tests in text were performed on non-normalized values. Lines are best fit to data collapsed across experimental condition.

This relationship is examined in Figure 9, where we plot color constancy (blue squares) and variability (red circles) as a function of number of palette chips in the cube region. Cube region was calculated as a circle with its center defined by the average color match in the baseline condition and its radius defined as the average variability of color matches in the baseline condition. The number of palette chips will clearly increase with cube region, and this increase may also be non-uniform. We confirmed that a wide range of radius values yielded the same pattern of results. To aid in visualization, both constancy indices and variability values were normalized to their respective maxima, but statistical tests were completed on the non-normalized data.

Color constancy under a change in illumination was unrelated to palette density, either in the joint condition (filled blue squares, *p* = 0.84) or in the illumination condition (unfilled blue squares, *p* = 0.19). In contrast, there was a negative correlation between variability of color matches and palette density (red symbols), although this correlation was stronger in the joint condition (filled red circles, *r* = −0.59, *p* < 0.05) than the illumination condition (unfilled red circles, *r* = −0.48, *p* = 0.06). Thus, in regions of color space with fewer possible matches, observers chose matches that spanned a wider range of color space.

## 4. Discussion

The main goal of this paper was to compare the effects of background and illumination on color matches in real objects to the large body of data on background and illumination effects in more simplified scenes. We found that the effects of illumination on average color matches generalized well from 2D to 3D, while the effect of background did not. In addition, both manipulations affected variability of color matches. Below, we discuss both findings as well as the implications of the specific task we employed.

### 4.1. Average color matches

We found that color constancy across a change in the illumination was very good (Figure 5), with an average color constancy index of about 90%, although the degree of constancy varied with cube. This is consistent with previous results in both 2D and 3D scenes with ample cues to the illuminant (Shevell and Kingdom, 2008; Brainard and Radonjic, in press).

That surfaces surrounding a colored surface or test patch affect its appearance is a well-known phenomenon: simultaneous contrast or color induction has been widely reported in a variety of different stimulus configurations (Shevell, 1978; Ware and Cowan, 1982; Chichilnisky and Wandell, 1995; Rinner and Gegenfurtner, 2002; Hurlbert and Wolf, 2004). Explanations of such background effects typically invoke some form of local contrast-coding, such as von Kries adaptation (Brainard et al., 1993). An implicit assumption is that a color constant visual system ought to attribute changes in the background color signal to a change in illumination, rather than a change in background reflectance. In simulated or simplified scenes, such as the classical patch/surround display, color signal changes are ambiguous. However, in real scenes where such color signal changes are in fact due to reflectance changes, local contrast is not a valid cue to surface reflectance; in such circumstances, constancy indices are typically much lower, around 20%, (Delahunt and Brainard, 2004; Allred and Brainard, 2009; Brainard and Radonjic, in press). An important question is whether such background effects, endemic in 2D scenes or for flat test stimuli and backgrounds embedded in 3D scenes, also exist for 3D objects and backgrounds.

The extent to which scene geometry affects color constancy is currently a topic of active research (Boyaci et al., 2003, 2004; Bloj et al., 2004; Delahunt and Brainard, 2004; Ripamonti et al., 2004; Boyaci et al., 2006; Allred and Brainard, 2009; Xiao et al., 2012). Though early work focussed on the importance of color contrast in determining color perception, more recent research has emphasized the importance of scene geometry. For example, Gilchrist demonstrated that the apparent lightness of a constant luminance test patch is influenced heavily by the depth and associated illumination with which it is grouped (Gilchrist, 1977).

The principle governing such reasoning is that the visual system segregates the scene into objects and regions of illumination/frameworks, and then applies color or lightness-mapping rules within each framework (for different theoretical implementations of these general principles, see Adelson, 2000; Gilchrist et al., 1999). Perceptual organization is thus of crucial importance: in this view, failures of constancy in classic simultaneous contrast illusions result from the failure of the visual system to segregate a test object from its surround or the incorrect assignment of a test stimulus to the appropriate region of illumination. From the anchoring/framework perspective, then, we might expect that any cues that increase the accuracy of object segregation or illuminant estimation would increase color constancy.

In contrast with the large body of research on flat, matte stimulus collections, we found that embedding a test cube in a 3D background had little effect on average color matches: errors in the background condition were similar to the split-half error in the baseline condition and background errors were significantly smaller than illumination errors (Figure 6). Further, in contrast to previous research (Delahunt and Brainard, 2004; Allred and Brainard, 2009), we also found that adding a background change to an illuminant shift (joint condition) did not substantially reduce color constancy indices (Figure 6). Thus, our data are consistent with the principles of anchoring or framework theories which postulate that local contrast cues can be silenced when the visual system is provided with sufficient evidence for perceptual segregation and illuminant estimation.

### 4.2. Variability of color matches

Generally, scene complexity is thought to improve color constancy (Shevell and Kingdom, 2008), although there are notable exceptions (see Foster, 2011, for discussion). Under one view of color constancy, scene complexity is postulated to do so by increasing the accuracy of the illuminant representation. Under this view, the visual system arrives at a reflectance estimate by combining a variable estimate of the illuminant (either implicitly or explicitly) with the incoming sensory signal (see Brainard and Maloney, 2011, for review). In such a view, failures of constancy are interpreted as mis-estimations of the illuminant. Although color constancy research typically focuses on the extent of average mis-estimation under the rubric of color constancy, it may be that an illumination shift also alters the overall uncertainty in the illuminant representation, and this could manifest itself as increased variability in color matches as well as the more traditionally reported decreased constancy. Although past research has generally focused on average constancy, a growing body of research seeks to understand the relationship between variability of responses and average responses in both color (Rinner and Gegenfurtner, 2000; Hillis and Brainard, 2005; Abrams et al., 2007; Hillis and Brainard, 2007a,b) and other visual domains (Weiss et al., 2002; Stocker and Simoncelli, 2006).

Two features of our data are consistent with this view. First, we found that the variability of color matches increased in the illumination condition (Figure 7). If illuminant estimation is indeed involved in achieving constancy in this task, then the illumination condition required observers to estimate the illuminant in both booths; this presumably increased uncertainty compared to the baseline condition. We also found an increase in matching errors in this condition (Figure 6).

Second, embedding a cube in the background decreased variability compared to the baseline condition (Figure 7). To understand this, consider that overall errors in this condition were relatively low, similar to split-half errors in the baseline condition (Figure 6). This suggests that the 3D cues present in the scene, cube, and background allowed the visual system to successfully segregate the background from the cube. If this is the case, then the background could be thought of as another nearby object in the scene that allows a second estimate of the same illuminant, thereby reducing the overall uncertainty in the illuminant estimation within the booth and the subsequent variability in the color matches. This view is further supported by noting that variability in the joint condition, where the background is added to the illumination shift, is less than in the illumination alone condition (Figure 7).

Interestingly, although conditions with higher constancy overall also tend to have less variability, we failed to find any within-condition correlations between color constancy for individual cubes and variability of color matches for that cube.

Although we have cast our interpretation of average constancy and variability in terms of illuminant estimation, we note that the available evidence suggests that observers do not explicitly represent the illuminant (Rutherford and Brainard, 2002; Amano et al., 2006; Granzier et al., 2009). Despite this, the language of illuminant estimation implicit in discussions of perceptual segregation may be functionally useful. Still, we note that there are alternative interpretations of our data for those reluctant to view perceptual segregation as either critically important or theoretically useful. For example, our scenes are relatively rich scenes with non-uniform illumination; thus, the local contrast relationships are more complex than they are in uniformly illuminated, 2D scenes. Previous work has suggested that with such information, low-dimensional linear models are in theory able to unambiguously recover both surface reflectance and illumination without resorting to higher level perceptual segmentation (Zmura and Iverson, 1994). However, such low-dimensional models have not yet been able to successfully predict human color judgments (see Foster, 2011, for dicussion).

### 4.3. Task

The discretized matching task employed here is very different than many other color constancy tasks. Many studies employ asymmetric matching, where observers adjust a matching stimulus under a test illuminant until it appears to match a standard under a standard illuminant (Kuriki and Uchikawa, 1996; Brainard et al., 1997; Faul et al., 2008; Kulikowski et al., 2012) or achromatic adjustment, where observers adjust the stimulus until it appears gray (Brainard, 1998; Boyaci et al., 2004; Hansen et al., 2006). Although some studies have employed discretized palettes, they typically use Munsell chips or papers (McCann, 2004; Olkkonen et al., 2010; Allred et al., 2012) or NCS papers (Hedrich et al., 2009). Such color spaces and palettes are used because they are thought to uniformly sample perceptual color space, and thus avoid potential artifacts due to uneven stimulus sampling.

Such palettes and tasks have proved fruitful in explaining laboratory color matching. However, palettes encountered in the real world, such as thread, fabric, or paints, are unlikely to uniformly sample color space. In addition, typical laboratory tasks often involve appearance matches, and there is considerable debate both about whether and when such matches may differ from reflectance matches (Troost and de Weert, 1991; Bäuml, 1999; Ripamonti et al., 2004; Brainard and Radonjic, in press). We chose here to focus on reflectance matches because they arguably underlie many behaviorally important tasks (Zaidi et al., 1992; Allred, 2012; Brainard and Radonjic, in press), but we acknowledge that others may have a different perspective on the functionality of appearance judgments.

With respect to these concerns, two of our findings are particularly relevant to the task demands. First, color constancy indices in the illumination condition were very similar to those reported in a variety of other studies employing relatively realistic stimuli, but using different tasks. Our observers were instructed to make a reflectance match; the nature of the task also supports a reflectance identification strategy. Furthermore, the lack of correlation between palette density and color constancy suggests that, at least for average constancy indices, the palette choice is not critical. Together, these findings provide support for the common assumption that the results from asymmetric matching and achromatic adjustment tasks in simulated scenes will generalize to more complex scenes and more realistic tasks.

Although the concordance between our findings and previous studies are encouraging, we recognize that several complications may arise from using a non-standard color task and palette. First, if the color palette is insufficiently discretized, then constancy indices could be artificially inflated. However, observers chose many different palette chips. On average there were 7.7 chips chosen for the 11.3 observers per cube. The raw number of paint chips chosen per cube was much higher than in some other studies using discretized chips (Hedrich et al., 2009), indicating that insufficient discretization is likely not a potential confound.

Second, non-uniformity of the matching palette makes it difficult to compare variability of color matches between cubes. For example, the greater variability in color matches for red than green (see Figure 7) could result either from more perceptual variability or from a less densely sampled palette. Indeed, we reported a negative correlation between palette density and variability of color matches (Figure 8). Since there was no correlation between palette density and average color constancy (Figure 9), palette non-uniformity is less likely to affect the interpretation of constancy for individual cubes.

## 5. Conclusions

As noted in the introduction, there are two broad classes of approach as we seek to move from relatively simple, parametrically manipulated stimuli and tasks to the full complexity of realistic scenes. One approach takes incremental steps, predicting and then testing the effect of manipulating one particular stimulus aspect such as object slant (Bloj et al., 2004) or cues to depth (Werner, 2006). Here we took the complementary approach of utilizing as realistic a scene and task as possible. We do not view our data as endorsing a specific theoretical view or mechanistic model of constancy; rather, we have the much more modest goal of providing some empirical constraints as we elaborate further theories of color vision. Our results suggest that average color constancy across illumination should remain high but variability should increase. Furthermore, the addition of a background either with or without an illumination change should introduce relatively few errors in average matches and should decrease matching variability. However, there are several limitations to our approach that caution against over-generalization.

First, although our stimuli and matching palette were real, relatively rich scenes, many real world scenes contain variables that our scenes did not. For example, real objects may not be uniformly colored, or they may contain textures or specular highlights that provide additional information to the visual system. Second, although the illumination varied within booths, real-scenes may have both abrupt and gradual illumination changes, and may vary over many orders of magnitude greater than ours (Xiao et al., 2012). Third, although observers performed an identification task with a real matching palette, the matching palette was not 3D. In some real-world identification tasks, observers often have additional cues such as shape that combine with color to guide behavior. Fourth, we note that although we chose a wide variety of cube and background colors, (Figures 1, 3) we did not parametrically manipulate either. As noted previously, there is a complex and sometimes contradictory literature surrounding the magnitude and direction of expected simultaneous contrast or color induction effects (see Ekroll and Faul, 2012, for discussion). Although in aggregate we found no effect of background, certain cube/background pairs (e.g., dark green) had higher error indices, and it remains possible that there is a subset of stimuli where backgrounds would have a larger effect. Lastly, we focused solely on reflectance judgments, and the distinction between appearance and reflectance judgments may be of particular importance in scenes like those used here. For example, it is clear from visual inspection of the cubes that each face of the cube appears different in some way, even though it is also easy to see that the cube is uniformly painted.

Taken together, these points suggest caution against over-generalization of our results. An important avenue for future research is to determine the relative importance of each of these factors in the constraining our ability to generalize from color matching in simplified laboratory tasks to the color tasks faced by individuals in everyday experience.

### Conflict of interest statement

The authors declare that the research was conducted in the absence of any commercial or financial relationships that could be construed as a potential conflict of interest.
